# Can Monitoring Make It Happen? An Assessment of How Reporting, Monitoring, and Evaluation Can Support Local Wellness Policy Implementation in US Schools

**DOI:** 10.3390/nu13010193

**Published:** 2021-01-09

**Authors:** Lindsey Turner, Yuka Asada, Julien Leider, Elizabeth Piekarz-Porter, Marlene Schwartz, Jamie F. Chriqui

**Affiliations:** 1 College of Education, Boise State University, Boise, ID 83725, USA; 2 Division of Community Health Sciences, School of Public Health, University of Illinois Chicago, Chicago, IL 60612, USA; yasada2@uic.edu; 3 Institute for Health Research and Policy, University of Illinois Chicago, Chicago, IL 60608, USA; jleide2@uic.edu (J.L.); epiekarz@uic.edu (E.P.-P.); jchriqui@uic.edu (J.F.C.); 4 Division of Health Policy and Administration, School of Public Health, University of Illinois Chicago, Chicago, IL 60612, USA; 5 Rudd Center for Food Policy and Obesity, Department of Human Development and Family Sciences, University of Connecticut, 1 Constitution Plaza, Hartford, CT 06103, USA; marlene.schwartz@uconn.edu

**Keywords:** child nutrition, school district, wellness policy, legal epidemiology, implementation

## Abstract

US school districts participating in federal child nutrition programs are required to develop a local wellness policy (LWP). Each district is allowed flexibility in policy development, including the approaches used for policy reporting, monitoring, and evaluation (RME). The aim of this convergent mixed-methods study was to quantitatively examine RME provisions in policies among a nationally representative sample of districts in the 2014–2015 school year in order to examine whether policies were associated with RME practices in those districts, and to qualitatively examine perceived challenges to RME practices. Data were compiled through the School Nutrition and Meal Cost Study and the National Wellness Policy Study. In multivariable regression models accounting for demographics, survey respondents were significantly more likely to report that their district had informed the public about LWP content and implementation, if there was a relevant policy provision in place. Having a strong policy (as compared to no policy) requiring evaluation was associated with reports that the district had indeed evaluated implementation. Having definitive/required provisions in policies was significantly associated with actual use of RME practices. RME activities are an important part of policy implementation, and these results show that policy provisions addressing RME activities must be written with strong language to require compliance. In interviews with 39 superintendents, many reported that RME activities are challenging, including difficulty determining how to monitor and show impact of their district’s wellness initiatives. Furthermore, the qualitative results highlighted the need for vetted tools that are freely available, widely used, and feasible for districts to use in assessing their progress toward meeting the goals in their LWPs.

## 1. Introduction

Changing practices in organizations typically requires efforts to “make it happen” rather than “letting it happen” [1]. School districts are one type of organization that has a large potential to impact the environments in which millions of US children and adolescents spend time, as nearly 50 million students are enrolled in approximately 100,000 public K-12 schools across the nation [2], where they typically spend 180 days of the year [3]. Policy change is often valuable in promoting system-wide changes [4,5,6], and policy interventions have been acknowledged as an important part of efforts to improve children’s health by creating health-promoting school environments [7,8]. Nearly all K-12 public schools and districts in the US participate in child nutrition programs (i.e., school meal programs) administered by the United States Department of Agriculture (USDA) [9,10], making nearly all of the 13,000 school districts nationwide subject to policy requirements issued by that federal agency. Since the 2006–2007 school year, all districts participating in the USDA child nutrition programs have been required to develop and implement a local wellness policy (LWP), which is a written document to “guide a school district’s efforts to establish a school environment that promotes students’ health, well-being, and ability to learn by supporting healthy eating and physical activity” [11]. In 2010, the Healthy, Hunger-Free Kids Act [12] further recognized the potential of LWPs and the need for additional efforts to improve school-level implementation of those policies; as a result, the USDA updated the requirements for these policies, issuing the Local School Wellness Policy Final Rule in 2016 [13].

A key aspect of the LWP final rule was to promote a more active process of implementation through attention to policy reporting, monitoring, and evaluation (RME). Districts are required to designate one or more officials as wellness policy leaders who are responsible for ensuring compliance; superintendents or assistant superintendents are commonly identified for this task. LWPs must include provisions about how the district designee will: (1) report to the public on the policy’s content; (2) measure the policy implementation and compliance at the school level every three years; and (3) share the results of such assessments with the public. In addition to other requirements, all LWPs must: (1) establish goals for nutrition promotion and education, physical activity, and other activities to promote wellness; (2) ensure that schools follow nutrition guidelines for all foods and beverages available at school, consistent with the USDA’s school meal standards [14] and Smart Snacks in School nutrition standards [15]; and (3) ensure that food and beverage marketing meets Smart Snacks standards. While districts are required to address these topics in written policy, each district is allowed considerable flexibility in how to write their own LWP, including what nutrition and physical activity goals to set, which tools and mechanisms to use for monitoring implementation at the school level, and how to evaluate progress toward the goals set forth in the LWP. While adaptability can generally be valuable in promoting the successful implementation of interventions [16], it is unclear whether this flexibility for districts to develop their own approach is helpful in facilitating LWP implementation.

Consistent with the adage that “what gets measured gets done”, many efforts to promote implementation have incorporated routine monitoring and evaluation as a core strategy [6]. For example, measurement-based care is considered an evidence-based best practice for providing health services in clinical settings [17] and schools [18], and assessment and monitoring are also essential for universal prevention programs [19,20]. Although not typically described as such, LWPs are a type of universal prevention program because their aim is to improve schoolwide environments for all students, not just those already at risk of adverse health outcomes due to poor nutrition and physical inactivity. While most school districts now have LWPs in place [21], and LWPs have generally increased in strength and comprehensiveness over time [21,22], there are still many areas of need for improving written LWPs, and accelerating their implementation. How LWPs are worded matters, because stronger LWPs are more likely to be implemented [23], and emerging evidence suggests that stronger and more-comprehensive LWPs are associated with better student behavioral outcomes (fruit/vegetable consumption, and physical activity) and weight outcomes [24].

Survey data from directors of 518 school food authorities (SFAs) in the 2014–2015 school year showed that while nearly all (99%) had an LWP, most had not evaluated it (64%) [25], although RME has been required since the Healthy, Hunger-Free Kids Act of 2010, and establishing a plan for measuring implementation of the LWP has been a required wellness policy component since the 2006–2007 school year. This leads to important questions about who is complying with wellness policy requirements (i.e., which districts are using monitoring), and why districts are or are not doing so. Thus far, almost no work has explored RME activities specific to LWP implementation, and no work has examined whether written policy provisions are associated with RME activities. Given superintendents’ critical oversight of district policy monitoring and evaluation, it is important to understand how they have engaged with such activities, and yet no work has done so. This convergent mixed-methods study combines a quantitative examination of RME policies and practices with a qualitative exploration of the perspectives of superintendents about RME activities. The aim of this manuscript is to examine the prevalence of policy provisions regarding RME, to examine associations between policies and actual RME practices, and to qualitatively examine perceived challenges to such practices in US school districts.

## 2. Materials and Methods

### 2.1. Data and Design

The written LWP data reported here were gathered as part of the National Wellness Policy Study, a convergent mixed-methods project to examine the implementation of LWPs. The current study utilized a sequential expansion approach [26], with primary and secondary quantitative data collection and analysis, followed by qualitative data collection conducted with the aim of further understanding the relationships assessed in the quantitative data. The secondary quantitative data source was the School Nutrition and Meal Cost Study (SNMCS), which was conducted in the 2014–2015 school year for the USDA [25]. Through collection of data from a nationally representative sample of school food authorities (SFAs), schools, and students, the SNMCS provides information on school food environments. The current study utilized the SFA Director Survey from SNMCS. The survey-weighted SNMCS data are nationally representative of public SFAs that offer the National School Lunch Program. The SNMCS methodology report details the design, sampling, recruitment, data collection, and data processing procedures [25]. Additional details about the data used in this paper are available in Volume 1 of the SNMCS report [27].

To create a dataset that includes LWP coding and all of the SNMCS data, the National Wellness Policy Study conducted primary policy data collection in the school districts that corresponded to the SNMCS nationally representative sample of SFAs, collecting and coding each district’s LWP and associated documents. Policy data were linked by Mathematica Policy Research based on district identifiers, and de-identified data were returned to the authors for analysis. This study was deemed to “not involve human subjects” by the University of Illinois Chicago Institutional Review Board (protocol #2020-0448).

The qualitative component of the National Wellness Policy Study [28] included stakeholder focus groups and interviews with food service directors, superintendents, students, and parents, with a goal of examining perspectives about wellness, and stakeholder experiences with LWP implementation. The current study uses superintendent data to complement the quantitative findings linking policies and practices. Additional findings from the superintendent studies are described elsewhere [29,30]. This study was approved by the University of Illinois Chicago Institutional Review Board (#2015-0720).

### 2.2. Measures

#### 2.2.1. SNMCS Outcome Measures

The SNMCS sample included 633 SFAs, 548 (86.6%) of which agreed to participate in this study; the SFA Director Survey was completed by 518 of those SFAs (95.7% weighted response rate). Four items from the survey were used in the current analyses (verbatim wording is shown below and in results). Three items were part of a block to assess “whether the component is addressed in your district wellness policy and, if so, the extent to which the wellness policy components have been implemented”. Those three components were: “Plan for informing the public about the wellness policy content and implementation” (reporting); “Plan for describing the progress made towards attaining the goals of the policy” (monitoring); and “Plan for measuring implementation of the policy, including the extent in compliance with the policy” (evaluation). The response metric for all three components was: (a) addressed in policy and fully implemented; (b) addressed in policy and partially implemented; (c) still being planned; or (d) not addressed in policy. As a second measure of SFA self-reported practices regarding evaluation, we used an additional survey item asking “Has your district ever evaluated the effects of the wellness policy”, with responses of yes or no.

#### 2.2.2. Local Wellness Policy Measures

Among the 518 SFAs in SNMCS with responses on the SFA Director Survey, we were able to obtain district policies from 496 of those districts (96%). The entities from which policies were obtained are hereafter referred to as “SFAs/districts”. The LWP was defined to include the board-adopted wellness policy; associated administrative regulations, rules, or procedures; and other district, state, or model policies that were incorporated by reference. Policies were relevant if in effect on the day after Labor Day, 2014, a proxy for the start of the 2014–2015 school year to enable linkage with the SNMCS survey data collected in spring 2015.

Policies were reviewed and verified by two members of the NWPS team, then coded by two trained analysts. Coding utilized an adaptation of a wellness policy coding scheme originally developed by Schwartz and colleagues [31] and modified by NWPS [32]. District policies were coded for whether three types of RME provisions were definitively required; recommended (encouraged, suggested, or required with exceptions); or not addressed in the LWP. Coding was grade specific, but generally did not vary across grade levels for the variables herein, except in one case each for R16 and E5 (described below), where the maximum coding across grade levels was used for analyses.

Public reporting of the policy (R1, R2) was assessed with two items regarding public posting or access to the LWP through either: (1) a website, or (2) a non-website location (e.g., posted in cafeteria or shared via newsletter). Strong policy provisions were those that were definitively required and specified an implementation plan or strategy, with language such as shall, must, will, require, comply, and enforce. Weak policies were those where reporting was suggested but not required.

Reporting on WP goals (R16) indicated whether the district required reporting on progress toward meeting WP goals. Strong policies had language definitively requiring reporting; examples were statements such as “The committee shall report on the status of compliance by individual schools and progress made in attaining goals established in the policy”, or “The superintendent shall report on the corporation’s compliance with this policy and the progress toward achieving the goals set forth herein when requested to do so by the board”. Weak policies had vague language, or reporting was recommended, but not required.

Plan for evaluation (E5) was coded to indicate whether the policy mentioned a plan for evaluation, including designating a person or group responsible for tracking outcomes. Strong policies included three elements: (1) an evaluation plan; (2) a person or group responsible for tracking evaluation; and (3) specific outcomes to be measured (i.e., health impact, student learning, School Health Index). Weak policies implied but did not specify the type of assessment, or did not specify whom is responsible for conducting the evaluation. Policies that mentioned “monitoring” without details were coded as having no provision.

#### 2.2.3. Contextual Characteristics

SFA/district characteristics were obtained from sources including the National Center for Education Statistics and the SFA Verification Summary Report 2012–2013 [33,34,35,36]. SFA size was categorized as <1000, 1000–5000, or >5000 students; district locale was categorized as urban, suburban, rural, or township based on the urban-centric locale codes from the National Center for Education Statistics [34]. The district child poverty rate was based on the 2011 Census Bureau Small Area Income and Poverty Estimates school district file [36], categorized as ≥20% versus <20%. The district racial/ethnic distribution was categorized as predominantly (≥66%) non-Hispanic White, majority (≥50%) non-Hispanic Black, majority (≥50%) Hispanic, and other [37]; Census region was categorized based on Census definitions as South, West, Midwest, and Northeast [38].

### 2.3. Study Sample

Among the 496 SNMCS SFAs where the SFA Director Survey was completed and we were able to obtain local wellness policy data, three were missing demographic data on one or more of the district characteristics needed for analyses, yielding an analytical sample of 493 SFAs. These SFAs/districts were located in 46 states and the District of Columbia. Of the characteristics shown in Table 1, the only significant difference between SFAs included or not included was for SFA size (the SFAs with missing data were smaller). Sample sizes for specific analyses varied from 488 to 490 due to item-specific missing data. Due to differences in the analytical sample, descriptive statistics in this paper may differ from those in the SNMCS report [27].

### 2.4. Quantitative Data Analysis

Analyses were conducted in Stata (version 13, StataCorp LP, College Station, TX, USA; 2016) and accounted for the survey design and analytic weights. First, descriptive statistics were computed to describe sample demographic characteristics and outcomes. Thereafter, a series of multivariable logistic regression models were computed at the SFA/district level to examine whether there were differences in the prevalence of SFA directors reporting compliance with each of the four key outcomes. Each model included the corresponding district LWP provision as a key predictor variable, while controlling for demographic covariates. Adjusted prevalences were computed from these models, which represent the average probability of SFAs/districts complying with each practice, while accounting for all other variables in the model.

### 2.5. Qualitative Data Analysis

Superintendent perspectives and experiences with RME were obtained through focus groups conducted at the annual meeting of The School Superintendents Association (AASA) in March 2017. Additional details are described elsewhere [29,30]. Six focus group sessions were conducted with 39 superintendents of public K-12 school districts, from 22 US states. A focus group guide was developed and included questions about awareness of LWPs, oversight and evaluation of LWPs, technical assistance and resources, perceived benefits and barriers, and food and beverage marketing policies. Focus groups lasted approximately 60 min. Focus group participants were invited to be contacted for follow-up key informant interviews, which were conducted between May and July 2017 with 14 of the 39 superintendents (interviews lasted 40–60 min). Focus groups and interviews were digitally recorded, transcribed verbatim, and team coded in ATLAS.ti software, Version 8 (Berlin, Germany). Two analysts met weekly to discuss coding discrepancies, emergent themes, and analytical issues and progress; themes related to RME were developed using principles of constant comparative analysis.

## 3. Results

### 3.1. Characteristics of SFA and District Sample, and Characteristics of Superintendent Sample

Table 1 shows the survey-weighted characteristics of the sample of SFAs/districts, which were spread across regions of the US, and from a range of locales although nearly half (45.3%) were from rural areas. Just over one-third of SFAs/districts (39.5%) served relatively high-poverty communities. Table 1 also presents demographics of the superintendent sample, which included participants from across the country, representing districts with varied demographic characteristics. With regard to LWP characteristics in this sample of SFAs/districts (not shown in tables), generally they did not address the public posting of the LWP on the district’s website, with 5.1% requiring it and 4.0% recommending it (90.9% did not address it). However, provisions regarding public posting of the LWP in locations other than the website was more common, with 12.5% of districts requiring it and 6.6% recommending it (80.9% did not have a provision). Provisions regarding monitoring whether the district had met LWP goals was required in policy for 29.6% of districts, and recommended in 2.2% (not addressed by 68.2%). Finally, the majority of districts had a policy provision about plans for LWP evaluation, with 19.7% requiring it, 51.8% recommending it, and 28.5% having no provision.

### 3.2. Prevalence of SFA-Reported Activities to Report, Monitor and Evaluate Wellness Policies

Next, the responses on the SNMCS SFA Director Survey were examined. As shown in Table 2, approximately one-third of SFAs/districts reported having fully implemented RME strategies, but more than one-third had either not addressed these activities at all or were still planning how to conduct RME activities. On the survey item asking whether the SFA/district had ever evaluated the effects of the LWP, 36.2% of respondents replied yes, but 63.8% had not (not shown in tables).

### 3.3. Policy Association with District/SFA Reporting, Monitoring and Evaluation Activities

A series of multivariable logistic regression models were calculated to examine associations between SFA/district RME activities and relevant policy provisions, while accounting for covariates. Table 3 presents summary information for associations between policy and each relevant outcome. All regressions included demographic covariates. Due to the low prevalence of policies about reporting LWP implementation progress on the district’s website (5.1% required it and 4.0% recommended it), these codes were combined as “any” policy. Having any policy was significantly associated with survey respondents indicating that their district/SFA had fully implemented a plan for informing the public about LWP content and implementation, as compared to districts without a policy (adjusted prevalences = 65.41% vs. 33.58%, *p* = 0.011). Similarly, strong policies about reporting results to the public through mechanisms other than the website were also significantly associated with survey reports that the SFA/district had fully implemented a plan for informing the public about LWP content and implementation: strong policies were significantly different from no policy (adjusted prevalences = 55.44% vs. 32.89%, *p* = 0.011). With regard to monitoring, having a policy provision requiring reporting on progress made toward meeting LWP goals was significantly associated with survey respondents indicating that their SFA/district had implemented plans to describe progress made toward attaining LWP goals: strong policies were associated with more monitoring, compared to no policy (adjusted prevalences = 44.84% vs. 29.02%; *p* = 0.028). Finally, regarding evaluation, having a strong policy requiring plans for evaluating the LWP was associated with SFAs/districts reporting that they had measured implementation of the policy (i.e., evaluating outcomes), compared with no policy (adjusted prevalences = 57.45% vs. 24.02%; *p* < 0.001). We also considered whether policy provisions regarding evaluation were associated with SFA survey respondents indicating that evaluation had occurred. SFAs/districts with a strong policy provision requiring evaluation were, indeed, significantly more likely to have evaluated the effects of the policy compared to those with no policy (adjusted prevalences = 55.41% vs. 26.47%; *p* = 0.006).

### 3.4. Qualitative Results: Perspectives from Superintendents about Reporting, Monitoring, and Evaluation

Superintendents, as school district leaders, are a critical stakeholder to gain insight into RME practices related to LWPs. Overall, superintendents in this study reflected that RME activities mostly posed challenges in their districts. Many explained that it was challenging to identify how to monitor and show impact of wellness initiatives, due to the complexity of the changes and observed outcomes across the district. Superintendents identified optimal technical assistance and support strategies that they would like to have to facilitate their RME activities going forward.

#### 3.4.1. Theme 1: Determining Causality Is a Challenge

Superintendents discussed the challenge of identifying measurement systems to determine the outcomes of implementing LWP initiatives. While participants were interested in understanding and measuring change, they often struggled with how to do so:

“…what’s the benefit of this policy and practice? We’ve had conversation about that and we really haven’t collected data. We feel like every time we are trying to decide whether that made a difference academically, we look back and go ‘yeah but we made these other 18 different changes.’ So we haven’t really found a way to assess whether or not an improvement… other than maybe physical health, but not from an academic standpoint.”

—Superintendent from Texas

“I think it’s really hard to put it as causation but when you start to look at rising test scores and there are a lot of things, I think it’s one of the things affecting that. It’s one factor. We can’t say ‘this caused that,’ but we can say we know we’re improving and as we’ve been working on implementing a wellness policy.”

—Superintendent from Alabama

Despite the stated challenges, participants offered examples of the types of data/indicators they had collected to evaluate LWP efforts. Some utilized established surveys while others collected data unique to their own initiatives.

“Our district participates every other year in the Youth Risk Behavior Survey, YRBS, so we have that data and we put that into our strategic plan as one of our key indicators.”

—Superintendent from North Dakota

“We’re beginning to collect qualitative data [referring to a mindfulness initiative] that it is having significant causative impact on student’s focus, conduct, building culture in classrooms.”

—Superintendent from Pennsylvania

#### 3.4.2. Theme 2: The Need for Tools and Resources

Superintendents were asked what would facilitate their RME activities and responded in two parts. First, research linking positive outcomes from wellness initiatives to academic outcomes was desired. Participants responded that they and their colleagues were most interested in the relationship with academic performance, but they often did not have the resources or skills to assess it. Second, superintendents mentioned the need for “vetted” resources to evaluate their wellness initiatives. Participants reported an interest in tools that were reputable and would be able to demonstrate the impacts of their wellness initiatives.

“If there was a tool we could use that had been vetted, that would help us evaluate our wellness program, that would be easy to administer to principals and teachers and provide reasonable data to help us gauge over the course of 5 years to say….we’re making a difference, we’re not making a difference….and where can we make changes.”

—Superintendent from Arizona

#### 3.4.3. Theme 3: How Leadership Facilitates Change

As we note elsewhere [30], superintendents can facilitate LWP implementation by translating the policy into common messages and creating a district-wide vision for wellness initiatives. Such coordination helps to indicate to other district and school staff that wellness initiatives are a priority for district leaders. This was described by one participant as “putting feet to the policy”, with efforts to improve implementation by identifying areas where the policy had not yet been implemented:

“I see my role as being able to show people the gap between what our policy says and our actual practice. Helping us find ways to close that. So, celebrating what we are doing well but also finding the one or two priority areas we need to work on further.”

—Superintendent, focus group

## 4. Discussion

Prior research shows that strong provisions in LWPs are associated with implementation of healthy nutrition standards at schools within those districts [23,24], and interventions such as mobilizing school-level wellness teams can improve the implementation of LWPs [39]. In the current study, rather than examining implementation of nutrition-specific standards, we examined several key process activities that are designed to accelerate the implementation of improvements in school nutrition environments; that is, reporting, monitoring, and evaluation activities. With a substantial volume of work on “how implementation happens” showing the importance of process strategies such as monitoring and evaluating progress, as well as reporting that progress back to key stakeholders [1,6,17,18,19,20], a focus on exploring these strategies is worthwhile. The Healthy, Hunger-Free Kids Act of 2010 [12] recognized the need for additional efforts to improve school-level implementation of LWPs. With the final LWP rule issued in 2016 [13], the USDA required that LWPs formally address RME activities. The current work explored whether such provisions in LWPs—either recommending or requiring that the SFA/district engage in RME activities—were associated with reports that such practices were actually occurring in those districts.

The survey data showed that, overall, in the 2014–2015 school year, RME activities were still relatively uncommon in SFAs/districts across the country. Only one-third of SFA directors reported already having implemented strategies to measure and evaluate progress toward their district’s wellness goals, or to have reported to the public on their LWP content, goals, and progress in implementing those changes. While it is likely that the prevalence of RME activities in districts across the nation has increased since the 2014–2015 school year, particularly with updated attention in the final rule [13], there are currently no nationally representative data since the 2014–2015 school year assessing whether districts have increased their use of RME activities to speed LWP implementation.

As described in the SNMCS report [27], the extent of SFA/district self-reported compliance with wellness policy components was high where the given components were present and evaluated, with mean scores (on a 5 point scale, where 5 = in compliance) of 4.6 for physical education, 4.4 for providing daily physical activity outside of physical education class, 4.5 for limiting access to competitive foods during school hours, 4.4 for nutrition education, and 4.5 for nutrition promotion. However, it is important to note that those high levels of compliance with LWP implementation occurred in only a fraction of districts, because the majority of SFA survey respondents indicated that although their district had an LWP they had not evaluated it (64%). In other words, districts that are evaluating their policies tend to be doing well with implementation, but for a majority of districts, it is unclear whether they are implementing the LWP goals well because they are not monitoring or evaluating progress.

The current work sought to explore whether written policy provisions in LWPs might be one leverage point for increasing districts’ use of RME practices, with the ultimate aim of increasing LWP implementation through better use of effective process strategies. The regression models did indeed show that LWP language requiring the district to monitor progress in meeting LWP goals was significantly associated with survey respondents noting that their district had engaged in such activities. The same was noted for policy provisions regarding evaluation activities, with evaluation occurring in 55% of SFAs/districts with a strong policy provision requiring evaluation, as compared to only 26% of SFAs/districts without such LWP language. Importantly, RME practices were more common in SFAs/districts where relevant policies were written with strong language, requiring action, whereas policies written as recommendations were not associated with significant increases in RME activities (as compared to SFAs/districts with no policies). In other words, having RME provisions seems promising for increasing districts’ use of RME activities to improve LWP implementation, but only when the language is definitive, rather than simply encouraging action.

One often-used approach in LWP development is the use of standardized or model wellness policy language. However, it appears that many state board model policies are written to ensure compliance with the law, and not to promote evidence-based best practices. Several studies have found that LWPs based on model policies are not necessarily stronger or more comprehensive than those that were locally developed [40,41]. For example, a study of 130 districts in VA found that locally-developed LWPs were both stronger and more comprehensive than policies based on templates from a state board, particularly for topics of LWP communication and promotion, and evaluation of the LWP; however, policies from both sources tended to be weak and lacking comprehensiveness [41]. Recent work in 2019 examined language in model wellness policies that had been issued by 34 states across the US [42], and found that, overall, the strength of the language for RME provisions was relatively low, with an average score of 21.0 (SD = 23.6), based on coding on the 100-point strength score from the WellSAT 3.0 [43], which is similar to the coding used in the current work. These findings highlight the importance of writing strong and comprehensive model policies.

Capturing the perspectives of multiple stakeholders is crucial for understanding how to improve the implementation and sustainability of system changes [44], and the current study incorporated perspectives from superintendents—the stakeholders most often held responsible for compliance with federal policies, such as the LWP rule. The qualitative data showed that superintendents found RME activities challenging. Many explained that they struggled to identify how to best monitor and show impact of their wellness initiatives, due to the complexity of the changes across the district. While they were interested in measuring impacts, they discussed the challenge of identifying measurement approaches to determine outcomes of wellness-related initiatives. Limited capacity seems to be a likely cause of the lack of district engagement in RME activities that was reflected in the survey data. Capacity is crucial for the implementation of any system change, and for monitoring in particular it can be challenging to build capacity to conduct monitoring or evaluation. Interviews with state nutrition directors in 2006—early in the process of LWP development—showed most (89%) perceived that districts had minimal capacity to monitor and evaluate their LWP [45]. Based on the qualitative data, this limited capacity—in terms of limited knowledge, tools, and expertise in monitoring/evaluation—remains challenging, and thus is a crucial topic to address if RME activities are to become more widespread.

The qualitative data showed that many superintendents perceived a need for vetted resources to evaluate initiatives, and an interest in reputable tools that can demonstrate impact. While a variety of tools exist for monitoring implementation of policies [46], most are research-focused and often are not accessible to practitioners. A recent review of observational tools for measuring school nutrition and physical activity environments identified 23 tools, but noted that many under-report usability indicators such as practicality, quality, and applicability, and may not be ideal for school/district personnel [47]. The School Health Index is a tool designed to assess school practices [48], and the WellSAT-I (www.wellsat.org) is an interview that matches each WellSAT 3.0 item with a question for the relevant school system representative (e.g., food service director, principal, teacher).

One of the challenges in monitoring LWP implementation is a disconnect between the individual responsible on paper, and the individual who must face state auditors. In most policies, the superintendent is listed as the individual responsible for compliance; however, the district food service director is the staff member who is monitored during the triennial compliance review by the state agency that oversees school nutrition programs [49]. Including superintendents in the triennial review process may engage these stakeholders who are crucial in the process of implementation and evaluation.

### Limitations and Areas for Future Research

Several limitations are notable. The quantitative data are cross-sectional, so it is not possible to infer causality between policies and practices. Further, there may be some bias as these are self-reported survey data. We note that there was not a perfect match between the language in the policy items and the practices assessed by the survey, and that some readers may consider there to be conceptual overlap between the practices of “monitoring” and “evaluation”. Future quantitative research should examine the extent to which policies impact subsequent use of RME strategies at both the district level and the school level. With regard to the qualitative data, the majority of superintendents were already interested in wellness; however, these participants were not meant to be representative, but rather to provide richer information about the perspectives of leaders. Future work may examine the perspectives of superintendents at districts that are not actively involved in prioritizing wellness. Seeking insights from superintendents in districts that either are—or are not—using RME strategies to disseminate the LWP, as well as objective information about progress toward implementation, may be valuable. Lastly, the sample of superintendents was not drawn from the SNMCS districts, thus there is not overlap between the two samples and we are unable to triangulate superintendent accounts of implementation with actual practices regarding RME, nor with changes in school wellness environments. The findings from superintendents point to potential reasons for the slow uptake of RME strategies/activities in districts observed in our quantitative findings. Superintendents are most often responsible for leading RME activities, yet their challenges with identifying the best assessment strategies (e.g., what to measure) and tools demonstrate a need for more targeted technical assistance to this specific stakeholder. In addition, because district food service directors are often held responsible for policy compliance, additional work is needed to consider their perspectives about RME activities.

## 5. Conclusions

The USDA’s final LWP rule allows flexibility for districts to develop policies that are appropriate for their specific contexts, needs, and resources. Prior work has shown that policies that are developed based on model wellness policies are not necessarily stronger or more comprehensive than those that are locally developed. However, and importantly, the current results emphasize the importance of LWP development that addresses key elements of the dissemination and implementation process, such as reporting, monitoring, and evaluation. We found that having definitive/required provisions in policies was significantly associated with the actual implementation and use of RME practices in those districts. Policy provisions that are written as suggestions rather than recommendations are not sufficient; such polices must be written with strong language to require compliance. Qualitative results further illustrated areas where implementation challenges occur, particularly around the need for vetted tools that are freely available, widely used, and feasible for districts to use in assessing their progress toward implementing LWP provisions and meeting the goals set forth in their LWPs. Tools such as the WellSAT policy coding tool and companion WellSAT-I implementation measurement tool hold promise for meeting these gaps in the translation of policy to practice.

## Figures and Tables

**Table 1:** Sample characteristics.

Variable	SFAs %	Superintendents *n* (%)
District Race/Ethnicity of Students		
Predominantly (≥66%) White	65.4	25 (64%)
Majority (≥50%) Black	7.1	4 (10%)
Majority (≥50%) Hispanic	8.3	3 (8%)
Other Majority or Diverse	19.2	7 (18%)
District Locale		
Urban	12.8	3 (8%)
Suburban	21.5	21 (54%)
Township	20.5	6 (15%)
Rural	45.3	9 (23%)
District-Level Eligibility for Free/Reduced-Priced Meals		
≤33% of students		19 (49%)
>33% to 66% of students		8 (21%)
≥67% of students		7 (18%)
District Child Poverty Rate		
<20%	60.6%	
20% or greater	39.5%	
SFA Size		
Small (<1000 students)	45.4	8 (21%)
Medium (1000 to 5000 students)	40.0	20 (51%)
Large (>5000 students)	14.6	11 (28%)
Region		
West	16.1	6 (15%)
Northeast	18.1	15 (38%)
South	25.5	7 (18%)
Midwest	40.3	11 (28%)

Notes: District and SFA characteristics were obtained from the National Center for Education Statistics, the 2011 Census Bureau Small Area Income and Poverty Estimates school district file, and the SFA Verification Summary Report 2012–2013. SFA percentages are survey-weighted, superintendent percentages are not weighted. *n* = 493 SFAs and *n* = 39 superintendents. SFA: school food authority.

**Table 2:** Prevalence of SNMCS survey-reported SFA activities to report, monitor, and evaluate local wellness policy implementation.

SFA Activity	SFA Activity Status (% of SFAs)			
	Fully Implemented	Partially Implemented	Still Being Planned	Not Addressed
Reporting: Plan for informing the public about the wellness policy content and implementation	36.4	26.0	30.2	7.4
Monitoring: Plan for describing the progress made towards attaining the goals of the policy	33.5	27.7	30.0	8.8
Evaluation: Plan for measuring implementation of the policy, including the extent in compliance with the policy	34.0	30.0	28.3	7.6

Notes: *n* = 488–489 SFAs included in the analytical sample due to item-specific missing data. SFA: school food authority; SNMCS: School Nutrition and Meal Cost Study. SFA Activity column reflects the verbatim SNMCS wording for each question.

**Table 3:** Summary of five logistic regression models to examine whether district-level policy is associated with SFA activities to report, monitor, and evaluate policy implementation.

	Adjusted Prevalence (% SFAs)	Adjusted Odds Ratio (95% CI)
Model 1: Reporting		
Outcome: Plan for informing the public about the wellness policy content and implementation		
Policy Predictor: Reporting to Public via Website (R1)		
No Policy	33.58	Referent
Any Policy	65.41	4.20 (1.40, 12.61) *
Model 2: Reporting		
Outcome: Plan for informing the public about the wellness policy content and implementation		
Policy Predictor: Reporting to Public, Non-Website (R2)		
No Policy	32.89	Referent
Recommended	42.81	1.57 (0.34, 7.20)
Required	55.44	2.69 (1.25, 5.76) *
Model 3: Monitoring		
Outcome: Plan for describing the progress made towards attaining the goals of the policy		
Policy Predictor: Reporting on meeting LWP goals and progress (R16)		
No Policy	29.02	Referent
Recommended	26.85	0.89 (0.11, 7.37)
Required	44.84	2.09 (1.09, 4.01) *
Model 4: Evaluation		
Outcome: Plan for measuring implementation of the policy, including the extent in compliance with the policy		
Policy Predictor: Plan for evaluating the LWP (E5)		
No Policy	24.02	Referent
Recommended	30.56	1.42 (0.73, 2.75)
Required	57.45	4.72 (1.98, 11.21) ***
Model 5: Evaluation		
Outcome: Has district ever evaluated the effects of the wellness policy		
Policy Predictor: Plan for evaluating the LWP (E5)		
No Policy	26.47	Referent
Recommended	34.59	1.51 (0.68, 3.34)
Required	55.41	3.80 (1.48, 9.77) **

Notes: All models included demographic covariates of region, SFA size, district locale, district race/ethnicity of students, and district child poverty rate (results not shown in table). Outcome variables are from the School Nutrition and Meal Cost Study (SNMCS) SFA Director Survey and were coded 1 = fully implemented, versus 0 = partially implemented, still being planned, or not addressed, except for model 5, where outcome was 1 = yes, 0 = no. *n* = 488–490 SFAs due to item-specific missing data. Local wellness policy (LWP) predictor variables were gathered and coded by the National Wellness Policy Study. SFA: school food authority; CI: confidence interval. * *p* < 0.05, ** *p* < 0.01, and *** *p* < 0.001.

## Data Availability

Requests for access to the public use SNMCS data should be submitted via electronic mail to: FNSStudies@usda.gov.
